# Persistent Hepatocellular Secretory Failure Secondary to Flucloxacillin-Induced Liver Injury: A Case With Successful Response to Rifampicin

**DOI:** 10.7759/cureus.95348

**Published:** 2025-10-24

**Authors:** Syed Muhammad Hussaini, Mohammad Adjmal Rummun, Vijeta Rummun, Ziyaulhaq Mustapha

**Affiliations:** 1 Internal Medicine, Blackpool Victoria Hospital, Blackpool, GBR; 2 Internal Medicine, East Lancashire Hospitals NHS Trust, Blackburn, GBR; 3 General Medicine, Blackpool Victoria Hospital, Blackpool, GBR

**Keywords:** cholestatic liver injury, drug induced liver injury (dili), flucloxacillin induced liver injury, hepatocellular liver injury, persistent hepatocellular secretory failure

## Abstract

We report the case of an 80-year-old male who developed severe cholestatic jaundice following two courses of flucloxacillin prescribed for a soft-tissue infection. Despite discontinuation of the antibiotic, serum bilirubin remained markedly elevated eight weeks later, accompanied by persistent pruritus. Comprehensive evaluation for acute liver injury, including viral, autoimmune, and metabolic studies as well as abdominal imaging, was unremarkable. A liver biopsy demonstrated preserved hepatic architecture with prominent hepatocellular cholestasis, consistent with hepatocellular secretory failure.

After consulting with a specialist hepatology centre, the patient was initiated on rifampicin monotherapy, resulting in gradual symptomatic improvement and a progressive decline in bilirubin levels over subsequent weeks. This case highlights the importance of maintaining a high index of suspicion for hepatocellular secretory failure in patients with persistent hyperbilirubinemia following withdrawal of a potential hepatotoxic agent. Targeted therapy with rifampicin can result in both symptomatic and biochemical improvement in hepatic function.

## Introduction

Persistent hepatocellular secretory failure is a rare but potentially life-threatening condition characterized by persistent dysfunction of hepatocytes in their capacity to secrete bile. When drug-induced, it represents a distinct phenotype of drug-induced liver injury (DILI), typically manifesting as profound and sustained jaundice that may persist for months following withdrawal of the offending agent. Unlike other forms of DILI, this entity is defined by the failure of bilirubin levels to normalize spontaneously despite cessation of the trigger. 

To date, rifampicin has emerged as a promising therapeutic option. Through complex biochemical mechanisms involving hepatic transporters and bile acid metabolism, rifampicin promotes a reduction in serum bilirubin over the course of several weeks.

Persistent hepatocellular secretory failure (PHSF) may be induced by drugs, toxins, or transient biliary obstruction. In certain cases, this dysfunction can persist for months following removal of the inciting factor and, if unresolved, may progress to fatal liver failure in the absence of liver transplantation [1]. More than 900 agents are known to cause liver injury, making drug-induced hepatotoxicity the most frequent reason for withdrawal of medications from clinical use [2]. This specific phenotype of drug-induced liver injury (DILI) is characterized by severe impairment of the hepatocyte’s ability to secrete bile and other essential substrates. 

The overall incidence of drug-induced hepatotoxicity in the general population has recently been estimated at approximately 14 cases per 100,000 inhabitants in Western countries, with drugs accounting for 10-52% of all cases of acute liver failure [3]. Data from the American Drug-Induced Liver Injury Network (DILIN) indicate that antibiotics are responsible for nearly half (45.4%) of DILI cases. Herbal and dietary supplements follow at 16.1%, representing a notable rise over the past 10 years, while cardiovascular agents (9.8%), central nervous system agents (9.1%), antineoplastic agents (5.5%), and analgesics (3.7%) are also common contributors [4]. 

Diagnostic criteria for hepatocellular secretory failure include serum total bilirubin (TBIL) > 255 μmol/L; persistent elevation of TBIL for more than one week following withdrawal of the offending factor; exclusion of obstructive cholestasis on imaging; and absence of pre-existing chronic liver disease [1]. 

An increase in serum bilirubin to more than twice the upper limit of normal, accompanied by elevated transaminases, signifies severe hepatotoxicity and carries a 10-15% risk of mortality if the offending agent is not discontinued (Hy’s Law) [5].

Rifampicin has been reported as the only effective pharmacological therapy for this condition. Functionally, rifampicin acts as a potent agonist of the pregnane X receptor (PXR), thereby inducing expression of cytochrome P450 3A4 (CYP3A4). CYP3A4 subsequently enhances bilirubin metabolism by promoting conjugation via enzymes such as UGT1A1, increasing the solubility of bilirubin and facilitating its clearance. In parallel, PXR activation induces the expression of multidrug resistance-associated protein 2 (MRP2), encoded by ABCC2, which mediates transport of bilirubin glucuronides into bile. Additionally, PXR upregulates other transporters, including P-glycoprotein, which promote elimination of detoxified bile acid metabolites through biliary or urinary excretion [6]. 

The clinical course of PHSF is variable, with some patients experiencing spontaneous resolution while others progress to end-stage liver failure requiring transplantation. To date, rifampicin remains the only reported medical treatment capable of significantly improving bilirubin clearance in affected individuals [7].

## Case presentation

**Figure 1:**
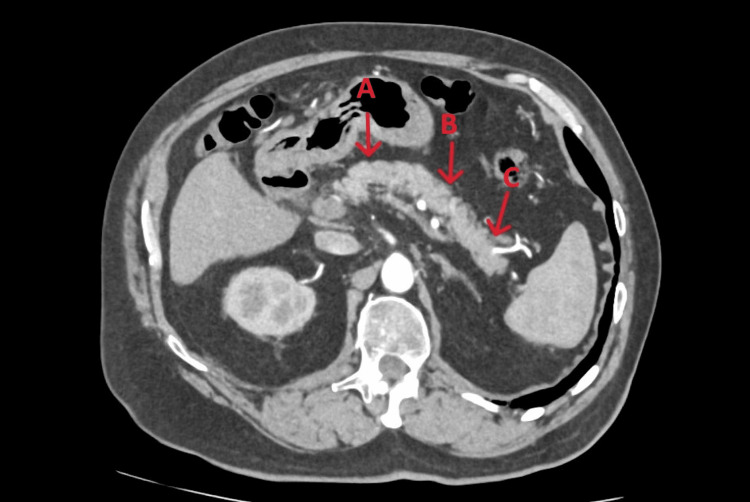
Computed Tomography (CT) pancreas showing normal tail/body without any pathology Arrows A and B indicate the body, and arrow C indicates the tail.

**Figure 2:**
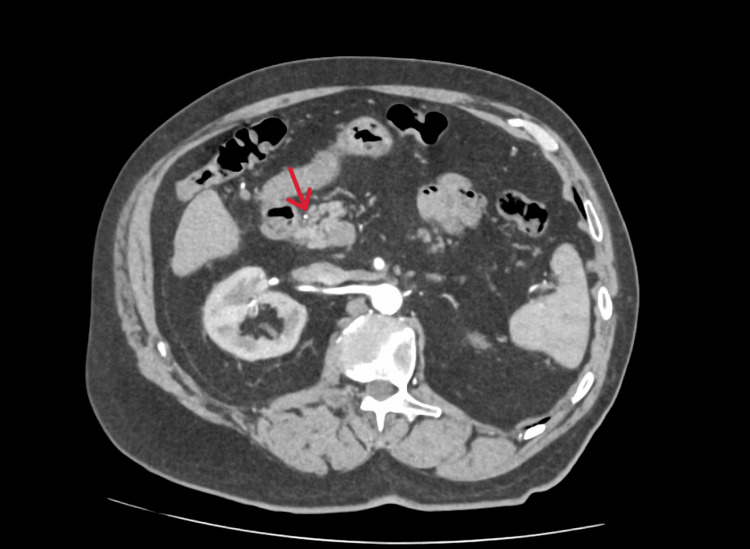
Computed Tomography (CT) Pancreas with the section of the head of pancreas showing no pathology The red arrow indicates the head of the pancreas.

An 80-year-old Caucasian man with a past medical history significant for atrial fibrillation, coronary artery disease, stage 3 chronic kidney disease (CKD3), hypertension, and hip osteoarthritis presented to his general practitioner (GP) on 29th of September 2025 after noticing scleral icterus, dark urine, and pale stools. He additionally reported unintentional weight loss of approximately 3-4 kg over the preceding year. Initial laboratory investigations revealed elevated serum bilirubin, prompting a computed tomography (CT) scan of the pancreas organised by the general practitioner, which took place on 4th of May 2025 as part of the pancreatic rapid diagnostic pathway, which revealed no pancreatic abnormalities but suggested a probable collapsed gallbladder containing gallstones (Figures 1, 2).

The patient presented to the Same Day Emergency Care (SDEC) unit on the 7th of May 2025 with worsening jaundice and associated pruritus. A decision was made to admit him for further evaluation and management.

The patient denied abdominal pain except for mild suprapubic discomfort. He also denied fever, chills, rigors, diarrhoea, recent travel, excessive alcohol intake, recreational drug use, or the use of herbal medications. His regular medications included rosuvastatin, ramipril, omeprazole, and warfarin; of these, rosuvastatin and warfarin were withheld due to deranged liver function. On further questioning, he reported having received two courses of flucloxacillin 500 mg four times daily for a soft-tissue infection approximately one month prior to the onset of jaundice.

On admission, the patient was visibly jaundiced. Physical examination revealed no abdominal tenderness, hepatosplenomegaly, ascites, or stigmata of chronic liver disease. Laboratory investigations performed by the general practitioner (GP) on the 29th of September 2025 showed elevated bilirubin, alanine aminotransferase (ALT), alkaline phosphatase (ALP), gamma-glutamyl transferase (GGT), and international normalised ratio (INR). Serum sodium, potassium, urea, and full blood count were within normal limits. Serum creatinine was 107 µmol/L, consistent with the patient’s baseline for known CKD3.

A comprehensive non-invasive liver screen, including iron studies, immunoglobulins, alpha-1 antitrypsin, caeruloplasmin, amylase, hepatitis A, B, C, and E serologies, cytomegalovirus (CMV) and Epstein-Barr virus EBV) IgM, leptospira IgM, and autoimmune markers (antinuclear antibodies (ANA), anti-smooth muscle, anti-mitochondrial, liver-kidney microsomal type 1 (anti-LKM-1) antibodies, soluble liver antigen, and antineutrophil cytoplasmic antibodies (ANCA)) was unremarkable.

**Figure 3:**
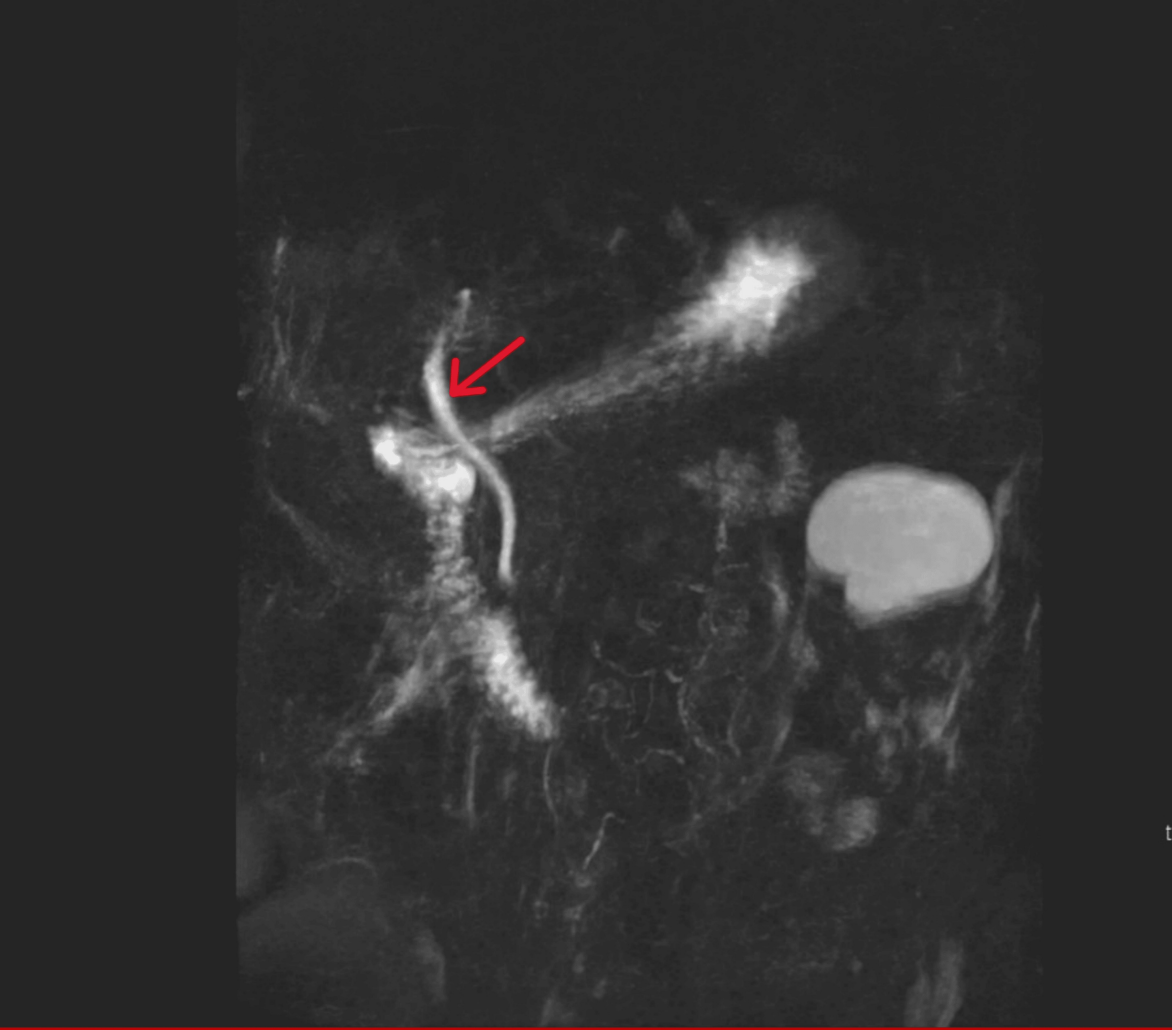
MRCP showing no biliary dilatation, no calculi within the CBD or cystic duct The red arrow indicates CBD. MRCP: magnetic resonance cholangiopancreatography; CBD: common bile duct

Further imaging, including abdominal ultrasonography on the 10th of May 2025, was unable to delineate the gallbladder and demonstrated no evidence of biliary dilatation. Magnetic resonance cholangiopancreatography (MRCP, Figure 3) and magnetic resonance imaging (MRI) of the liver, performed on the 14th of May 2025, did not identify a definitive cause for the patient’s jaundice.

Given the absence of an identifiable cause for the patient’s persistent jaundice and continuing rise in bilirubin, reaching a peak of 760 µmol/L, with an icteric ALP and ALT of 114 IU/L on the 20th of May 2025, despite supportive measures, a decision was made to refer him to a specialist hepatology and liver transplant centre for further evaluation. The hepatology team suspected hepatocellular secretory failure secondary to flucloxacillin-induced drug-induced liver injury (DILI) and recommended a liver biopsy for diagnostic clarification.

Around this time, the patient’s renal function also began to deteriorate, with progressively rising urea and creatinine levels. He was referred to the specialist nephrology team, who diagnosed acute kidney injury (AKI) on a background of CKD3, likely secondary to bile cast nephropathy. Their management recommendations included maintaining adequate hydration with intravenous fluids in the absence of signs of fluid overload, with the expectation that renal function would improve as bilirubin levels decreased.

**Figure 4:**
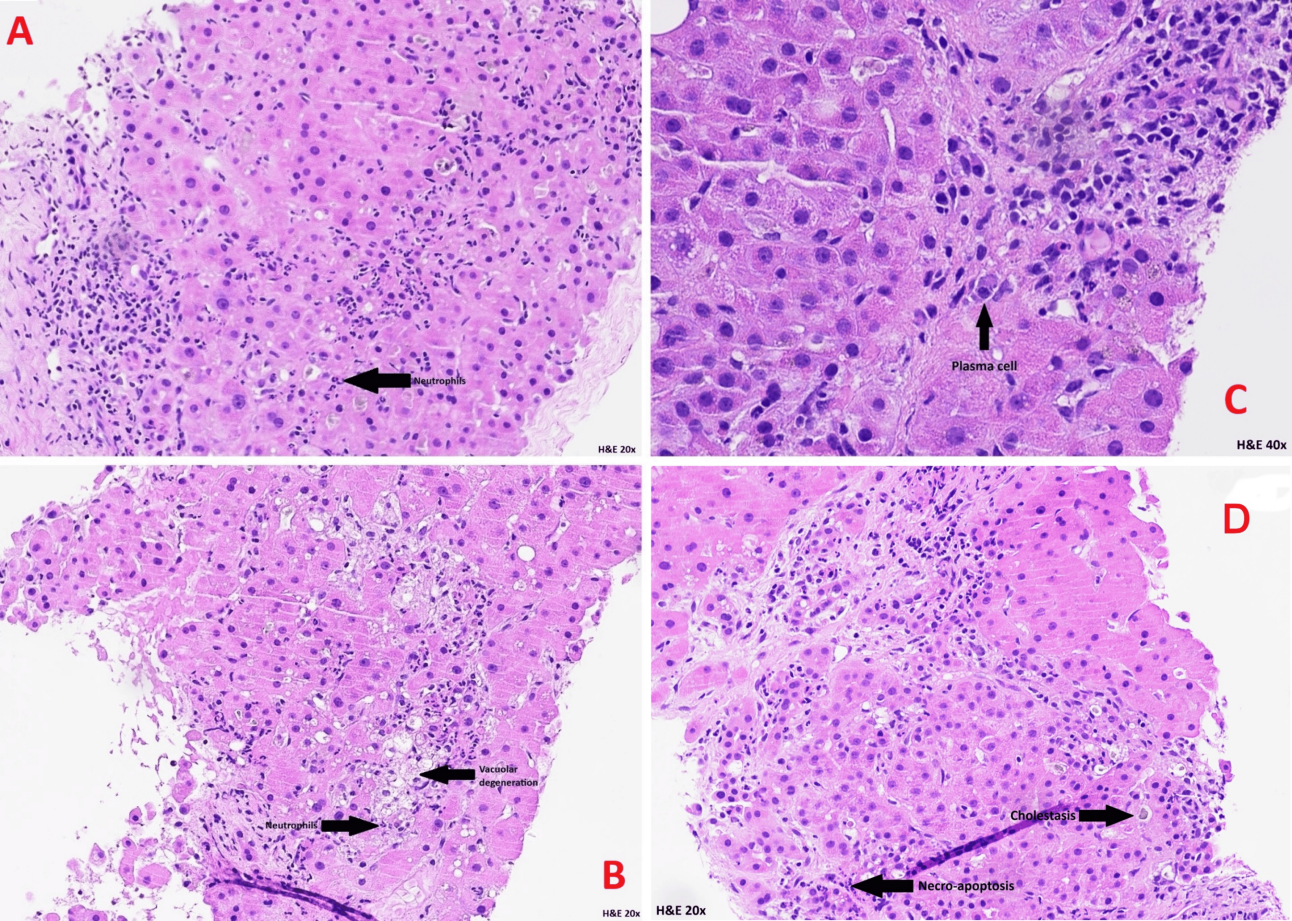
Histopathology The Portal tracts show a moderate increase in inflammatory cells, which are rich in neutrophils (A and B) as well as containing lymphocytes and plasma cells (C). The presence of a neutrophil-rich inflammatory cell infiltrate and prominent cholestasis is suggestive of drug-induced hepatitis. Cholestatic hepatitis with duct injury and duct loss consistent with flucloxacillin-induced liver injury (D).

A percutaneous liver biopsy was performed on the 19th of May 2025, and the initial histopathology (Figure 4A-4D) demonstrated features of acute hepatitis and biliary stasis. The specimen was subsequently referred to the specialist hepatology and liver transplant unit for further evaluation.

On the 4th of June 2025, the hepatology team initiated treatment with rifampicin 150 mg twice daily and prophylactic fluconazole 50 mg once daily to address presumed persistent hepatocellular secretory failure secondary to flucloxacillin-induced DILI.

Definitive histological analysis, reported on the 16th of June 2025 by the specialist hepatology and liver transplant centre, confirmed the diagnosis. The biopsy showed features of cholestatic hepatitis with bile duct injury and duct loss, accompanied by a neutrophil-rich inflammatory infiltrate and prominent cholestasis - findings consistent with flucloxacillin-induced liver injury.

**Figure 5:**
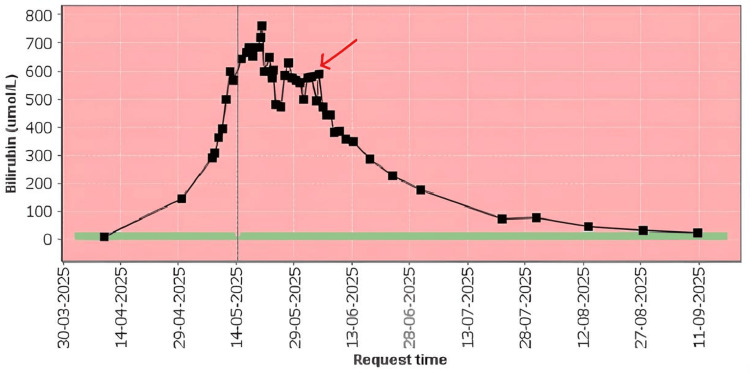
Trajectory of serum bilirubin levels during hospitalisation and following initiation of rifampicin therapy The red arrow indicates the commencement of rifampicin on the 4th of June 2025. This demonstrates an improvement trend following rifampicin and the return of bilirubin to the normal range.

The patient’s overall liver and renal function began to demonstrate gradual improvement following the initiation of treatment (Tables 1, 2), with a marked reduction in serum bilirubin levels over time (Figure 5).

**Table 1:** Serial biochemical and coagulation parameters prior to initiation of rifampicin INR: international normalized ratio; ALP: alkaline phosphatase; ALT: alanine aminotransferase; gamma GT: gamma-glutamyl transferase; icteric: sample too jaundiced to measure

Parameters	Initial bloods	On admission	Day 5	Day 10	Day 13	Day 17	Day 20	Day 25	Reference range
INR	–	9.0	1.2	1.3	1.2	1.4	1.2	1.2	
Urea	6.0	Icteric	Icteric	Icteric	Icteric	Icteric	Icteric	Icteric	2.5-7.8 (mmol/L)
Creatinine	107	108	120	124	147	274	281	225	64-104 (µmol/L)
Bilirubin	144	287	597	651	760	603	584	575	0-20 (µmol/L)
ALP	362	344	Icteric	Icteric	Icteric	Icteric	Icteric	Icteric	30-130 IU/L
ALT	235	105	88	97	114	106	98	106	0-49 IU/L
Gamma GT	801	–	623	–	–	–	–	–	0-54 IU/L

**Table 2:** Serial biochemical and coagulation parameters following initiation of rifampicin (commenced on 4th of June 2025) INR: international normalized ratio; ALP: alkaline phosphatase; ALT: alanine aminotransferase; gamma GT: gamma-glutamyl transferase; icteric: sample too jaundiced to measure

Parameters	4^th^ of June	Day 1	Day 2	Day 5	Day 20	Day 27	Day 48	2 months	27^th^ of August	Reference range
INR	1.2	1.2	1.4	1.2	1.4	1.3	–	–	1.1	
Urea	Icteric	Icteric	Icteric	Icteric	11.2	9.0	–	–	8.4	2.5-7.8 (mmol/L)
Creatinine	248	263	286	253	252	217	–	–	139	64-104 (µmol/L)
Bilirubin	588	472	445	383	224	177	73	78	30	0-20 (µmol/L)
ALP	Icteric	Icteric	Icteric	220	307	631	396	355	330	30-130 IU/L
ALT	93	79	68	64	51	96	29	41	44	0-49 IU/L
Gamma GT	–	–	–	–	–	–	–	–	–	0-54 IU/L

## Discussion

The clinical course of acute liver failure (ALF) typically begins with the onset of severe acute liver injury (ALI). This phase is defined by a two- to threefold elevation of serum transaminases, indicative of hepatocellular damage, accompanied by evidence of impaired hepatic function such as jaundice and coagulopathy, occurring in the absence of pre-existing chronic liver disease. Although this description was initially derived from cases of drug-induced hepatotoxicity, it remains applicable across the spectrum of ALF presentations [8] 

In patients presenting with drug-induced hepatocellular jaundice, the risk of progression to death or liver transplantation has been reported at 11.7%. Among implicated agents, amoxicillin-clavulanate represents the most frequently associated cause of drug-induced liver injury (DILI) [7]. Among the therapeutic groups of medications known to cause idiosyncratic liver injury, antibiotics rank first in terms of causative agent [9].

Real-world evidence regarding the use of rifampicin in cholestasis, particularly in patients with marked jaundice, remains limited. In a study conducted between 2016 and 2020, 14 of 16 patients demonstrated improvement in liver function tests - defined as a reduction exceeding 20% - at four weeks following initiation of rifampicin therapy [10]. These findings support the consideration of rifampicin in the management of the current case, where cholestasis has been demonstrated through histopathological findings from the liver biopsy, and a significant reduction in bilirubin was also evident in the data presented (Table 2 and Figure 6). 

Persistent hepatocellular secretory failure has been described in association with transporter gene mutations. In this context, rifampicin exerts its anticholestatic effect through activation of the pregnane X receptor (PXR), leading to induction of CYP3A4, UGT1A1, MRP2, and OSTβ [1]. This mechanism may account for the improvement observed in the present case, although genetic studies were not performed in this instance.

## Conclusions

This case illustrates a rare presentation of flucloxacillin-induced hepatocellular secretory failure complicated by bile cast nephropathy in an elderly patient, manifesting as severe, persistent cholestatic jaundice with delayed recovery despite discontinuation of the offending agent. The comprehensive evaluation, including imaging and extensive laboratory testing, may be unrevealing, and despite initial diagnostic uncertainty, timely specialist referral and liver biopsy remains a valuable tool for confirming hepatocellular cholestasis and guiding management.

Targeted therapy with rifampicin can lead to significant symptomatic and biochemical improvement of both hepatic and renal function. Clinicians should maintain a high index of suspicion for drug-induced liver injury in patients presenting with unexplained jaundice, particularly in those with recent antibiotic exposure. Early recognition, timely specialist referral, and multidisciplinary management are critical for optimizing outcomes.
